# Predicting Post-treatment HIV Remission: Does Size of the Viral Reservoir Matter?

**DOI:** 10.3389/fmicb.2021.648434

**Published:** 2021-02-26

**Authors:** Alexander O. Pasternak, Christina K. Psomas, Ben Berkhout

**Affiliations:** ^1^Laboratory of Experimental Virology, Department of Medical Microbiology, Amsterdam UMC, University of Amsterdam, Amsterdam, Netherlands; ^2^Department of Infectious Diseases and Internal Medicine, European Hospital, Marseille, France

**Keywords:** HIV, viral reservoir, antiretroviral therapy, post-treatment controllers, predictive marker, biomarker, profile

## Abstract

Combination antiretroviral therapy (ART) suppresses human immunodeficiency virus (HIV) replication and improves immune function. However, due to the persistence of long-lived HIV reservoirs, therapy interruption almost inevitably leads to a fast viral rebound. A small percentage of individuals who are able to control HIV replication for extended periods after therapy interruption are of particular interest because they may represent a model of long-term HIV remission without ART. These individuals are characterized by a limited viral reservoir and low reservoir measures can predict post-treatment HIV remission. However, most individuals with a low reservoir still experience fast viral rebound. In this Perspective, we discuss the possible reasons behind this and propose to develop an integral profile, composed of viral and host biomarkers, that could allow the accurate prediction of post-treatment HIV remission. We also propose to incorporate information on the chromatin context of the proviral integration sites into the characterization of the HIV reservoir, as this likely influences the reactivation capacity of latent proviruses and, together with the actual number of intact proviruses, contributes to the replication competence of the reservoir.

## Introduction

Combination antiretroviral therapy (ART) can successfully manage human immunodeficiency virus (HIV) replication but is not curative, due to the persistence of long-lived viral reservoirs (Deeks et al., 2016; Ndung’u et al., 2019). The main reservoir is thought to reside in latently infected resting CD4 + T cells in peripheral blood and lymphoid tissue, although other cell types such as macrophages may contribute as well (Darcis et al., 2019; Ganor et al., 2019). Even after many years of successful treatment, ART interruption typically leads to a viral rebound within 2–4 weeks; however, some individuals, termed “post-treatment controllers,” are able to control HIV replication after therapy interruption for extended periods and thus may represent a model of long-term HIV remission without ART or functional cure (Hocqueloux et al., 2010; Steingrover et al., 2010; Salgado et al., 2011; Goujard et al., 2012; Lodi et al., 2012; Van Gulck et al., 2012; Stöhr et al., 2013; Sáez-Cirión et al., 2013; Assoumou et al., 2015; Kinloch-de Loes et al., 2015; Frange et al., 2016; Maggiolo et al., 2018; Namazi et al., 2018; Violari et al., 2019). Therefore, recent years have seen accelerated research into mechanisms of HIV control in these rare individuals and in the macaque models of post-treatment control (PTC) (Strongin et al., 2020). Several excellent reviews on PTC have been published (Cockerham et al., 2016; Goulder and Deeks, 2018; Martin and Frater, 2018; Etemad et al., 2019), and a mathematical model of the underlying mechanisms has been proposed (Conway and Perelson, 2015). PTC is more frequent after ART initiated during early HIV infection (Namazi et al., 2018), which is not surprising given that a low viral reservoir has been consistently measured in post-treatment controllers (Goujard et al., 2012; Van Gulck et al., 2012; Sáez-Cirión et al., 2013), and early ART initiation is much more efficient in reducing the reservoir than ART initiated during chronic infection (Strain et al., 2005; Jain et al., 2013; Buzon et al., 2014). However, early ART initiation is not sufficient for PTC as most early treated individuals, even those treated very early after infection, still demonstrate fast viral rebound upon therapy interruption (Gianella et al., 2015; Colby et al., 2018). Longer ART duration has also been proposed to increase the chances of PTC (Stöhr et al., 2013; Fidler et al., 2017), but this factor on its own is insufficient to confer this phenotype. Identification of individuals with a higher probability of PTC, in whom it is safer to interrupt ART than in others, is of utmost importance in light of HIV cure research, where every therapeutic intervention necessitates an analytical treatment interruption (ATI) to assess its efficacy. A low risk of viral rebound during an ATI means low risks of reservoir replenishment, selection of drug resistance, disease progression, and HIV transmission (El-Sadr et al., 2006; Julg et al., 2019). The absence of reliable predictive markers of viral rebound complicates clinical decision-making on ATI and therefore hinders HIV cure research (Li et al., 2015). To fill this knowledge gap, a number of studies have been undertaken to identify biomarkers that could predict PTC or the time to viral rebound after ART interruption (Williams et al., 2014; Assoumou et al., 2015; Li et al., 2016; Sharaf et al., 2018; Pasternak et al., 2020). The latter measure may be more inclusive than the former, as PTC is a spectrum (Martin and Frater, 2018), and its definitions differ between studies, in particular in terms of the minimal duration of viral control and of the threshold for the viral rebound (Sáez-Cirión et al., 2013; Williams et al., 2014; Li et al., 2016; Namazi et al., 2018). Variable definitions of PTC also contribute to the variability in the frequency of post-treatment controllers between studies, which ranges from < 1% to > 20% and is inversely proportional to the duration of control (Hocqueloux et al., 2010; Sáez-Cirión et al., 2013; Maenza et al., 2015; Namazi et al., 2018). To standardize these definitions, Martin et al. proposed to reserve the term “PTC” for the cases of long-term continuous HIV control (several years) below the lowest possible detection limit of commercial plasma viral load assays (20 copies/mL) and to use the term “virological remission” for the intermediate cases that do not fulfill these strict criteria (Martin and Frater, 2018). Hence, using time to viral rebound as an outcome measure allows the inclusion of all these intermediate cases, increasing statistical power and potentially allowing additional insights into the mechanisms of control. On the other hand, some individuals who demonstrate various degrees of post-treatment HIV remission undergo transient viral rebound shortly after ART interruption before resuppressing the virus (Namazi et al., 2018), and measuring the time from ART interruption to the viral rebound will exclude these individuals. Clearly, improved definitions of post-treatment remission are needed to guide future HIV cure trials.

### Predicting Post-treatment HIV Remission: Time for a Comprehensive Approach

A number of candidate predictive biomarkers for the virological remission have been proposed. However, the measured markers, timing of their measurement, thresholds for viral rebound, and the statistical analyses that were performed differ significantly between studies. This, in combination with limited sample sizes, resulted in different and even some contradictory conclusions. Several studies identified total HIV DNA, measured just before ART interruption, as a predictor of time to viral rebound (Goujard et al., 2012; Williams et al., 2014; Assoumou et al., 2015), and this marker even outperformed the number of intact proviruses in distinguishing individuals with post-treatment virological remission from non-controllers in a recent study (Sharaf et al., 2018). On the other hand, three independent groups reported that cell-associated (CA) HIV unspliced RNA, measured at ART interruption, could predict time to viral rebound, while total HIV DNA was not predictive in these studies (Li et al., 2016; Sneller et al., 2017; Pasternak et al., 2020). It must be noted that CA RNA was not measured in most studies that did identify total DNA as a predictor, precluding a direct comparison between these markers. Importantly, we demonstrated that the pre-treatment-interruption level of CA unspliced RNA was predictive not only of the time to viral rebound to both >50 and >400 copies/mL but also of the magnitude of the viral rebound, independently of pre-ART virological biomarkers (Pasternak et al., 2020). This suggests that measurements of the “active reservoir” (Pasternak et al., 2013) or “transcription-competent reservoir” (Baxter et al., 2018) can help support the HIV cure-directed clinical trials (Abdel-Mohsen et al., 2020). However, standardization of assays and the CA RNA transcripts that are measured is warranted in order to obtain meaningful results. Apart from the unspliced RNA, PCR-based assays have been developed to measure levels of total, completed (polyadenylated), or multiply spliced CA HIV RNA transcripts in infected individuals (Pasternak et al., 2008; Shan et al., 2013; Yukl et al., 2018). The assay for total CA RNA uses primers that bind to the HIV TAR region and thus measures the level of transcription initiation. This TAR RNA is more abundant than unspliced RNA, but most of these transcripts are short and do not encode viral proteins (Lassen et al., 2004; Yukl et al., 2018). On the other hand, the presence of multiply spliced RNA may be a more proximal surrogate of productive infection compared with unspliced RNA only (Pasternak and Berkhout, 2018). However, multiply spliced RNA is much less abundant than unspliced (Kaiser et al., 2007; Pasternak et al., 2020), due to both proviral genetic defects (as splicing requires the presence of several intact genomic regions) and latency blocks to completion of transcription and splicing (Yukl et al., 2018; Moron-Lopez et al., 2020). As a consequence, it is challenging to detect multiply spliced RNA in ART-treated individuals without *ex vivo* cellular stimulation, which explains why it has not yet been assessed as a potential predictor of the post-treatment remission.

In addition to CA RNA, plasma HIV RNA blips on ART were also shown to predict shorter time to rebound (Fidler et al., 2017). Further studies in larger cohorts are necessary in order to establish whether the total number of HIV proviruses, HIV transcriptional activity, or a combination of these markers, can be used to support HIV curative interventions. Although total HIV DNA is mostly composed of replication-defective proviruses (Bruner et al., 2016; Hiener et al., 2017), and a significant proportion of CA RNA molecules might be transcribed from such defective proviruses as well (Pollack et al., 2017; Imamichi et al., 2020), both HIV DNA and CA RNA correlate with the inducible provirus levels (Darcis et al., 2017; Cillo et al., 2018), suggesting their utility as surrogate markers of the replication-competent HIV reservoir (Avettand-Fènoël et al., 2016; Pasternak and Berkhout, 2018). In any case, it is clear that a low viral reservoir is extremely important for HIV remission, and mathematical models have been developed that are based on the assumption that the duration of remission is inversely proportional to the replication-competent HIV reservoir size (Hill et al., 2014, 2016; Conway and Perelson, 2015; Davenport et al., 2019). It would therefore seem logical that ATI performed in a group of ART-treated individuals with low reservoir measures may result in HIV remission in a substantial proportion of cases. This has indeed been attempted by several groups (Chun et al., 2010; Calin et al., 2016; Colby et al., 2018; Pannus et al., 2020), but the absolute majority of cases experienced a quick viral rebound, suggesting that a low reservoir alone is insufficient for HIV remission and that other factors need to be considered. Here it must be noted that our understanding of the HIV reservoirs and their importance for the prediction of the post-treatment remission is still largely limited to the peripheral blood, whereas tissue reservoirs might play an even more important role. Different cellular and anatomical compartments, such as follicular T helper cells in the lymph node germinal centers, may serve as sanctuaries for HIV persistence under ART (Banga et al., 2016; Chaillon et al., 2020) and fuel the viral rebound upon ATI (De Scheerder et al., 2019). Although sampling peripheral blood is obviously easier, better characterization of tissue reservoirs can improve the predictive value of the HIV reservoir for the post-treatment remission.

In addition to virological markers, several host biomarkers have been proposed to predict post-treatment HIV remission. Pre-ART levels of T-cell exhaustion markers (PD-1, Tim-3, and Lag-3) have been shown to predict the time to viral rebound, although their on-ART levels were not predictive (Hurst et al., 2015). Two recent studies identified plasma and antibody glycomic biomarkers, in particular digalactosylated G2 glycans on IgG, as predictive markers of post-treatment remission (Giron et al., 2020; Offersen et al., 2020). Moreover, pre-ATI levels of HIV gp120-specific G2 glycans inversely correlated with CA HIV unspliced RNA levels (Offersen et al., 2020), providing a possible explanation why it was predictive of longer time to rebound. Although the role of cytotoxic T lymphocytes (CTLs) in post-treatment HIV remission is probably not as pronounced as in spontaneous (“elite”) HIV control and post-treatment controllers mostly lack protective HLA alleles (Goujard et al., 2012; Sáez-Cirión et al., 2013; Maenza et al., 2015), this does not mean that other components of the host immunity are not important. In fact, ART initiated extremely early, during the “hyperacute” HIV infection (Fiebig stage I), rarely results in prolonged post-treatment remission (Henrich et al., 2017; Colby et al., 2018), which is thought to reflect an insufficient time window for maturation of the adaptive immune responses (Goulder and Deeks, 2018). In contrast, the SPARTAC and Primo-SHM studies where temporary ART was started during primary infection, but not too early, resulted in some participants experiencing various degrees of post-treatment remission (Stöhr et al., 2013; Pasternak et al., 2020).

It therefore appears useful to develop a comprehensive molecular profile, incorporating multiple viral and host biomarkers, that could reliably predict post-treatment HIV remission. Such a profile could be based on the principle of diagnostic multivariate index assays that are already used in other medical fields (Zhang, 2012). The advantage of such a composite molecular profile, compared to single biomarker assays, is that the aggregated information from complementary biomarkers is expected to outperform each of the individual component biomarkers in sensitivity, specificity, and predictive value. Applied to the prediction of post-treatment HIV remission, such a profile may be composed of metabolomic, lipidomic, and proteomic biomarkers, in combination with virological and immunological profiling. In addition, the expression of recently identified cellular markers of the HIV reservoirs, such as CD32a, CD30, CD20, PD-1, and others (Fromentin et al., 2016; Descours et al., 2017; Abdel-Mohsen et al., 2018; Hogan et al., 2018; Serra-Peinado et al., 2019; Darcis et al., 2020; Neidleman et al., 2020; Adams et al., 2021), as well as T-cell phenotypic markers (Hiener et al., 2017), could be incorporated in this profile. Indeed, CD30+ CD4+ T cells, as well as expression of some HIV restriction factors, were shown to increase before viral rebound after ATI (De Scheerder et al., 2020; Prator et al., 2020). Furthermore, Mitchell et al. (2020) recently demonstrated that plasmacytoid dendritic cells can sense HIV replication before detectable viremia following treatment interruption, which was evidenced by a transient loss of IFNα production. Expression of cellular factors that are involved in long-term cell survival and proliferation vs. apoptosis could also play a role (Kuo et al., 2018; Angin et al., 2019). In this regard, as no single molecule has yet been described that marks all reservoir cells, a combinatorial approach will again be beneficial and perhaps even necessary. Ideally, the evolution of such a comprehensive molecular profile could allow the development of a personalized approach to HIV curative interventions. In particular, a gender-specific approach might be necessary, since several (but not all) studies demonstrated lower CA HIV RNA levels in women compared to men (Scully et al., 2019; Falcinelli et al., 2020; Gianella et al., 2020), and estrogen has been shown to repress HIV transcription (Das et al., 2018). Such approach should also include the personal medical history of each individual, namely the level of persistent immune activation despite ART, the history of comorbidities that is often associated with chronic inflammation, as well as current and historical ART regimens, all of which may contribute to the probability and timing of viral rebound.

### Post-treatment Remission and the HIV Reservoir Size: Are We Measuring the Right Markers?

Low HIV reservoir is necessary but apparently not sufficient for post-treatment remission, as even individuals with very low levels of reservoir markers experience fast viral rebound upon ART interruption. As discussed above, one possible solution to this problem is to identify other, complementary biomarkers, thus increasing the predictive power of the resulting profile. However, another possibility is that our current toolkit simply does not allow sufficiently accurate measurement of the HIV reservoir size. The latter is defined as the number of cells carrying replication-competent proviruses, in other words integrated viral genomes capable of reigniting viral spread upon ART interruption (Eisele and Siliciano, 2012; Pasternak and Berkhout, 2016). However, it is difficult to estimate the real HIV reservoir size, as PCR-based methods that measure HIV DNA and RNA overestimate the reservoir because most of proviruses are genetically defective (Bruner et al., 2016). On the other hand, the quantitative viral outgrowth assay (qVOA) will not score defective proviruses, but is thought to underestimate the reservoir as only a small fraction of genetically intact proviruses can be activated *ex vivo* (Ho et al., 2013; Bruner et al., 2016; Kwon et al., 2020; Martin et al., 2020). The most accurate surrogate marker of the reservoir size is currently considered to be the number of intact proviruses, estimated by either full-length proviral sequencing (Bruner et al., 2016; Hiener et al., 2017; Pinzone et al., 2019) or the recently developed digital droplet PCR-based intact proviral DNA assay (IPDA) (Bruner et al., 2019). Not all intact proviruses are replication-competent, as full-length sequencing is only able to identify gross genetic defects, such as large internal deletions, hypermutation, stop codons, frameshift mutations, or defects in the major splice donor site or the packaging signal, and will not identify other genetic changes that may be deleterious for HIV replication. However, most of the intact proviruses demonstrate normal replication kinetics *in vitro* (Ho et al., 2013), suggesting that the majority of proviruses identified as intact by full-length proviral sequencing are replication-competent. In comparison, IPDA overestimates the intact reservoir somewhat, as only ∼70% of proviruses that are identified as intact by IPDA are also intact by full-length proviral sequencing (Bruner et al., 2019).

There might be, however, another level of complexity to the measurement of HIV reservoir. By applying the novel multiple displacement amplification (MDA)-based matched integration site and proviral sequencing (MIPSeq) technique, the Lichterfeld group demonstrated that in individuals on prolonged ART, in comparison to defective proviruses, intact HIV proviruses were enriched for non-genic chromosomal positions and other features of “deep latency” (Einkauf et al., 2019). This bias was subsequently confirmed by another group that also used the MDA method (Patro et al., 2019). More importantly, the same technique, recently applied to the characterization of the HIV reservoir in elite controllers, revealed that this population demonstrates an even more extreme phenotype than ART-treated individuals: 40% of intact proviral clones in elite controllers were integrated into non-genic or pseudogenic regions, compared to 13% in ART-treated individuals (Jiang et al., 2020). Moreover, in contrast to ART-treated individuals, intact proviral sequences from elite controllers were preferentially integrated in centromeric satellite DNA or in other regions associated with heterochromatin, and at an increased distance to transcriptional start sites and accessible chromatin, and were enriched in repressive chromatin marks. As infection of CD4+ T cells from elite controllers *ex vivo* with a laboratory HIV strain led to a normal integration pattern, it is likely that this skewed integration pattern observed *in vivo* is the result of selective elimination of cells infected with transcriptionally competent intact proviruses over time by the immune system, resulting in enrichment for intact proviruses that are in a state of “deep latency” (also referred to as “blocked and locked” state) and are unlikely to be reactivated (Jiang et al., 2020). It was demonstrated 20 years ago that the provirus transcriptional activity is influenced by the integration site (Jordan et al., 2001), and indeed, levels of HIV transcription in elite controllers were shown to be at least 10-fold lower than in ART-treated individuals (Jiang et al., 2020), confirming the results of previous studies (Van Gulck et al., 2012; Hatano et al., 2013). Interestingly, Battivelli et al. (2018) found that HIV reactivation in a primary CD4+ T-cell model of latency occurred in at most 5% of the infected cells and depended on integration in an open chromatin context, which was confirmed by another group that demonstrated that inactive chromatin marks accumulate across the provirus with time (Lindqvist et al., 2020). The proportions of clonally expanded intact proviruses were shown to be larger in elite controllers than in ART-treated individuals (Veenhuis et al., 2018; Jiang et al., 2020), with the same pattern observed in a post-treatment controller (Veenhuis et al., 2018). Moreover, CD8+ T cells from the elite and post-treatment controllers were capable of suppressing replication of their autologous clonally expanded viruses *in vitro* (Veenhuis et al., 2018), suggesting that these intact proviruses can undergo clonal expansion without or with minimal viral gene expression (Hosmane et al., 2017; Musick et al., 2019).

**FIGURE 1:**
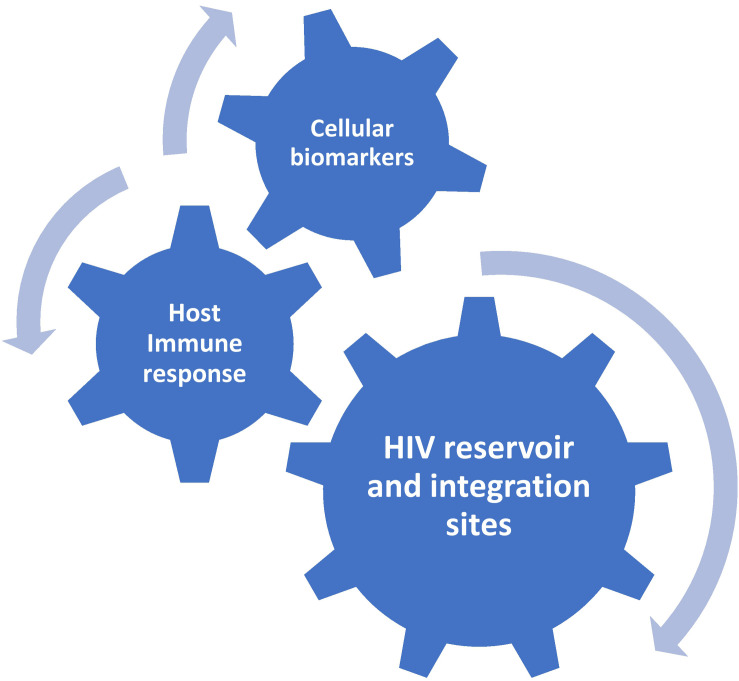
Proposed molecular profile for the prediction of post-treatment HIV remission.

Taken together, these recent insights imply that the integration site-imposed reactivation potential of a provirus could be as important as its genetic intactness (Chomont, 2020). In other words, not only the size of the reservoir, but also its repertoire (not only in terms of chromatin context but also in terms of diversity and clonality of proviral integration sites) matters for the replication competence. Moreover, they bring into doubt the concept that qVOA profoundly underestimates the replication-competent reservoir, providing a possible explanation why only a tiny fraction of intact proviruses can be reactivated *ex vivo*. On the other hand, Ho et al. reported that most non-induced intact proviruses in their study were integrated into active transcription units, suggesting that other factors exist that prevent intact provirus reactivation, at least *ex vivo* (Ho et al., 2013). Although the reactivation abilities *ex vivo* and *in vivo* cannot be directly compared, and there always is a possibility that a provirus that cannot become reactivated *ex vivo* even after multiple rounds of TCR stimulation, still can reignite viral rebound *in vivo* after ART interruption, we might consider the number of intact proviruses as the upper, conservative, limit of the replication-competent reservoir. In most infected individuals, the reservoir is probably much lower than this limit and in order to be able to accurately quantify the reservoir size, it would be necessary to combine the measurement of genetic intactness with that of *in vivo* reactivation potential, although measuring the latter is very difficult if not impossible. As a surrogate, a simple score based on the provirus intactness and the chromosomal context of its integration site could be developed, as MDA-based and other similar assays that simultaneously measure the provirus intactness and map the integration site provide the possibility to do this. Importantly, such a combined score may be able to predict time to viral rebound after ATI and/or post-treatment HIV remission better than other reservoir measures. To further improve the predictive power, this score could be incorporated into the molecular profile proposed above (Figure 1). Indeed, although the mechanisms of control are different between most elite and post-treatment controllers, studies have identified a subpopulation of elite controllers with both markedly inefficient *ex vivo* HIV reactivation from resting CD4+ T cells and low HIV-specific CD8+ T-cell responses (Noel et al., 2016; Canouï et al., 2017). This subpopulation may in fact resemble post-treatment controllers, in most of which no protective HLA alleles were found and CD8+ responses are also not particularly strong, and the virus replication is probably controlled due to infrequent reactivation from latency. Further research is needed to establish whether the HIV integration site landscape in post-treatment controllers resembles that of elite controllers (Jiang et al., 2020). This should involve longitudinal studies to evaluate immune selection for viral reservoir cells (Wang et al., 2018; Huang et al., 2019).

Finally, if the proviral integration sites are so important for the replication competence of the reservoir, is there something that can be done therapeutically to facilitate HIV integration in regions that are associated with the repression of transcription? HIV preferentially integrates in actively transcribed genes (Han et al., 2004; Marini et al., 2015) but long-term ART selects for transcriptionally silent proviruses (Pinzone et al., 2019). However, ART cannot do wonders and even after decennia of suppressive therapy, most individuals will experience a fast viral rebound upon ART interruption. Therefore, a number of strategies to “block and lock” the provirus in the inactive state are currently under investigation (reviewed in Moranguinho and Valente, 2020; Vansant et al., 2020a). In particular, the Debyser group developed a technique, based on the small-molecule (LEDGIN) inhibition of the interaction between the HIV integrase and its host cofactor LEDGF/p75, that allows retargeting HIV integration from active genes to sites that are less transcriptionally active, indeed resulting in lower HIV transcription (Vranckx et al., 2016; Vansant et al., 2020b). However, it is still unclear how this technique could be applied in infected individuals, as the HIV reservoir is formed very early after infection and once the provirus is integrated, it cannot be retargeted. Interestingly, several groups recently reported that in the untreated infection, the reservoir turns over quickly, and that most proviruses in ART-treated individuals match circulating HIV variants from shortly before ART initiation (Brodin et al., 2016; Abrahams et al., 2019; Pankau et al., 2020). In this case, treatment with LEDGINs or similar compounds shortly before the start of ART could indeed result in a lower transcriptional activity of the reservoir and, as a consequence, a higher frequency of post-treatment HIV remission.

## Conclusion

In summary, although a number of biomarkers are already identified that can predict post-treatment HIV remission, there are still major gaps in our understanding of its underlying mechanisms. Consequently, ATIs are still the only way to assess the efficacy of new HIV curative interventions, and criteria for the recruitment of clinical trial participants remain unclear. Further research is urgently needed to identify robust and validated predictive biomarkers of post-treatment remission. In this regard, the development of an integral biomarker profile as outlined above should facilitate the efforts to achieve prolonged virological control in the absence of ART.

## Data Availability Statement

The original contributions presented in the study are included in the article/Supplementary Material, further inquiries can be directed to the corresponding author/s.

## Author Contributions

AP wrote the first draft of the manuscript. CP contributed to the figure. All authors contributed to the writing and approved the final manuscript.

## Conflict of Interest

The authors declare that the research was conducted in the absence of any commercial or financial relationships that could be construed as a potential conflict of interest.
